# 

**Published:** 1877-03

**Authors:** 


### Books and Pamphlets Received.

Healthy Skin: A Popular Treatise on the Skin and Hair, their Preservation and management. By Erasmus Wilson, F. R. S., F. R. C. S., Eighth Edition. Philadelphia: Lindsay & Blakiston, 1876.

Chemical and Microscopical Examination of the Urine in Health and Disease. By Geo. B. Fowler, M. D. New York: G. P. Putnam's Sons, 1876. Buffalo: Peter Paul & Bro.

The Electric Bath: Its Medical Uses, Effects and Appliances. By Geo. M. Schweig, M. D. New York: G. P. Putnam's Sons, 1877. Buffalo: Peter Paul & Bro.

A Series of American Clinical Lectures. Edited by E. C. Seguin, M. D. Vol. II. New York: G. P. Putnam's Sons, 1877. Buffalo: Peter Paul & Bro.

Transactions of the American Medical Association: Twenty-seventh Annual Meeting, Philadelphia, June, 1876. Philadelphia: Printed for the Association.

Cyclopædia of the Practice of Medicine. Edited by H. Von Ziemssen. Vol. VI.: Diseases of the Chylopœtic System. New York: Wm. Wood & Co. Buffalo: H. Matteson, Agent.

Report on the Influence of Climate on Pulmonary Diseases in Minnesota. By Franklin Staples, M. D.; Reprint from Transactions of American Medical Association.

Fifth Annual Report of the New York Ear Dispensary. New York: G. P. Putnam's Sons, 1876.

The Operations of Vesico Vaginal Fistula; A Comparison of the Methods of Operating of N. Bozeman, M. D., of New York, and Prof. Gurtan Limon, of Heidelberg. With Illustrations. Translated from Weimer Woehenschift by A. C. Bernays, M. D. St. Louis: Reprint from Richmond and Louisville Medical Journal, 1877.

Tablets of Anatomy and Physiology. By Thomas Cooke, F. R. S., Eng., M. D. Anatomy Ear, Eye. Physiology; Part I. New York: Wm. Wood & Co., 1873.

Thirty-seven Operations for Thoracentesis by Pneumatic Aspiration. By Frank Donalson, M. D. Reprint from Transactions Medical and Chirurgical Faculty of Maryland. Baltimore: 1876.

### Contents of March Number.

### ORIGINAL.

**THE BUFFALO**

### Medical and Surgical Journal

**PUBLISHED MONTHLY,**

**Containing Original Articles, Reports of Medical Societies and Hospitals, Editorial, Reviews, Correspondence, Army News, etc., etc ; including the usual variety of Medical Periodical Publications. Specimen copies sent on application.**

Address Buffalo Medical & Surgical Journal, Buffalo, N. Y.

**TERMS OF SUBSCRIPTION, $3.00 PER YEAR, IN ADVANCE.**

